# Automated classification of coronary LEsions fRom coronary computed Tomography angiography scans with an updated deep learning model: ALERT study

**DOI:** 10.1007/s00330-024-11308-z

**Published:** 2025-01-10

**Authors:** Victor A. Verpalen, Casper F. Coerkamp, José P. S. Henriques, Ivana Isgum, R. Nils Planken

**Affiliations:** 1 Department of Radiology and Nuclear Medicine, Amsterdam University Medical Center, University of Amsterdam, Amsterdam Cardiovascular Sciences, Amsterdam, The Netherlands; 2 Department of Cardiology, Amsterdam University Medical Center, University of Amsterdam, Amsterdam Cardiovascular Sciences, Amsterdam, The Netherlands; 3 Department of Biomedical Engineering and Physics, Amsterdam University Medical Center, University of Amsterdam, Amsterdam Cardiovascular Sciences, Amsterdam, The Netherlands; 4 Faculty of Science, University of Amsterdam, Informatics Institute, Amsterdam, The Netherlands

**Keywords:** Artificial intelligence, Coronary computed tomography angiography, Coronary artery disease, Deep learning, Inter-reader variability

## Abstract

**Objectives:**

The use of deep learning models for quantitative measurements on coronary computed tomography angiography (CCTA) may reduce inter-reader variability and increase efficiency in clinical reporting. This study aimed to investigate the diagnostic performance of a recently updated deep learning model (CorEx-2.0) for quantifying coronary stenosis, compared separately with two expert CCTA readers as references.

**Methods:**

This single-center retrospective study included 50 patients that underwent CCTA to rule out obstructive coronary artery disease between 2017-2022. Two expert CCTA readers and CorEx-2.0 independently assessed all 150 vessels using Coronary Artery Disease-Reporting and Data System (CAD-RADS). Inter-reader agreement analysis and diagnostic performance of CorEx-2.0, compared with each expert reader as references, were evaluated using percent agreement, Cohen’s kappa for the binary CAD-RADS classification (CAD-RADS 0-3 versus 4-5) at patient level, and linearly weighted kappa for the 6-group CAD-RADS classification at vessel level.

**Results:**

Overall, 50 patients and 150 vessels were evaluated. Inter-reader agreement using the binary classification at patient level was 91.8% (45/49) with a Cohen’s kappa of 0.80. For the 6-group classification at vessel level, inter-reader agreement was 67.6% (100/148) with a linearly weighted kappa of 0.77. CorEx-2.0 showed 100% sensitivity for detecting CAD-RADS ≥ 4 and kappa values of 0.86 versus both readers using the binary classification at patient level. For the 6-group classification at vessel level, CorEx-2.0 demonstrated weighted kappa values of 0.71 versus reader 1 and 0.73 versus reader 2.

**Conclusion:**

CorEx-2.0 identified all patients with severe stenosis (CAD-RADS ≥ 4) compared with expert readers and approached expert reader performance at vessel level (weighted kappa > 0.70).

**Key Points:**

***Question*** *Can deep learning models improve objectivity in coronary stenosis grading and reporting as coronary CT angiography (CTA) workloads rise?*

***Findings*** *The deep learning model (CorEx-2.0) identified all patients with severe stenoses when compared with expert readers and approached expert reader performance at vessel level.*

***Clinical relevance*** *CorEx-2.0 is a reliable tool for identifying patients with severe stenoses (≥ 70%), underscoring the potential of using this deep learning model to prioritize coronary CTA reading by flagging patients at risk of severe obstructive coronary artery disease.*

## Introduction

Coronary computed tomography angiography (CCTA) is increasingly used as the initial diagnostic test in patients with stable symptoms suspected of coronary artery disease (CAD) [1–3]. The strength of CCTA is the ability to accurately rule out CAD with an overall sensitivity of 95% [4]. Current coronary artery stenosis grading, however, is predominantly based on human visual assessment, which is subject to inter-reader variability [5–7]. Standardized reporting with Coronary Artery Disease-Reporting and Data System (CAD-RADS 2.0) is used to decrease variability among CCTA readers and guide patient management decisions. The CAD-RADS score is a valuable tool for the classification of stenosis [8–10]. CAD-RADS includes six categories on a CAD-RADS 0 to CAD-RADS 5 grading scale (0%, 1–24%, 25–49%, 50–69%, 70–99%, and 100% stenosis) and a non-diagnostic category (CAD-RADS N). Despite this standardized CAD-RADS reporting, accurate CCTA reading requires a high level of expertise [7, 11]. Expert CCTA readers are expected to detect obstructive CAD (CAD-RADS ≥ 3) significantly more accurately than non-expert readers [6]. However, the number of expert readers is not unlimited, and the number of CCTA scans is rising rapidly [3, 12]. Therefore, the CCTA-related workload is expected to escalate. Artificial intelligence (AI) has increasingly been used for automated image analysis tasks, which may aid in facilitating timely reporting [13]. For quantitative measurements, the use of automatic deep learning-based models (DLM) may reduce observer variability and increase efficiency in CCTA reporting [14, 15]. Previous studies have shown promise of DLMs in automating the classification process and demonstrated good agreement with expert readers [16–20]. An earlier commercially available DLM (CorEx-1.0; Spimed-AI) showed a diagnostic accuracy of 96% at patient level using the binary CAD-RADS classification threshold of 50% stenosis (CAD-RADS 0-2 versus CAD-RADS 3-5) [20]. The aim of the current study was to investigate the diagnostic performance of a recently updated DLM (CorEx-2.0) for quantifying coronary stenosis on CCTA, using a binary CAD-RADS classification threshold of 70% stenosis (CAD-RADS 0-3 versus CAD-RADS 4-5) at patient level, as a threshold for potential interventional treatment, and using the 6-group CAD-RADS classification (CAD-RADS 0-5) at vessel level, compared separately with two independent expert CCTA readers as references.

## Methods

### Study design

This single-center study is not subject to the Medical Research Involving Human Subjects Act (WMO) as approved by the accredited institutional Medical Research Ethics Committee (MREC). We obtained written informed consent from all patients in accordance with the General Data Protection Regulation (GDPR). In total, 50 patients who underwent CCTA to rule out obstructive CAD were retrospectively included. All CCTA examinations were obtained in routine clinical practice at our center between 2017 and 2022. Patients were selected from a quality database, aiming to ensure a balanced CAD-RADS distribution and a substantial representation of CAD-RADS categories around the stenosis grade threshold of 50–69% diameter stenosis (CAD-RADS 2, 3, and 4). Patients with acute coronary syndrome, coronary stents, bypass grafts, or uninterpretable CCTA (CAD-RADS N assigned in clinical practice) were not included.

### CCTA scan acquisition

For all CCTA exams, a third-generation dual-source 192 detector row computed tomography scanner (Somatom Force, Siemens Healthcare) was used. Beta-blockers, including metoprolol 50 mg or 100 mg, were administered orally to reduce heart rate 1 h before the scan if the heart rate was > 65 beats per minute. Sublingual nitroglycerine spray was administered for vasodilatation. Before scanning, patients were asked to breathe normally preceding a breath-hold for scan acquisition. All scans were acquired in prospective sequential mode (step and shoot) and electrocardiogram triggered in preferentially late diastole. For the CCTA scans, automatic tube voltage selection (CARE kV, Siemens Healthcare) was applied in all patients with kV values ranging from 70 to 120 kV with increments of 10 kV. The time between the start of contrast medium injection and the time to peak contrast enhancement in the ascending aorta was determined using a test bolus injection with a fixed contrast bolus of 10 mL undiluted contrast medium (Ultravist 300: iopromide 300 mg I/mL, Bayer AG or Xenetix 350: iobitridol 350 mg I/mL, Guerbet OptiVantage DH) with an injection rate of 6.5 mL/s, a fixed scan delay of 8 s and a fixed kV value of 100 kV. For timing the CCTA acquisition, the scan delay was determined by the time to peak and an additional 4 s for coronary artery filling time. The contrast bolus for CCTA was adjusted to patient body weight and kV selection [21]. All CCTA scans were visually evaluated for diagnostic image quality by the attending CCTA technician immediately after the acquisition.

### CorEx CAD-RADS analysis

CCTA examinations were uploaded to SPIMED-AI and analyzed by the previously described CorEx version 1.0 (CorEx-1.0) and by the updated CorEx version 2.0 (CorEx-2.0) [20, 22]. In short, both models were trained using the Inception-v3 convolutional neural network (CNN) architecture developed by Google [23]. While keeping the same architecture, CorEx-2.0 was retrained on a new dataset with more diverse cases and a slightly different CAD-RADS distribution.

For the present study, axial CCTA images were uploaded to SPIMED-AI, and curved multi-planar reconstruction (cMPR) images were created for CorEx analysis. None of these CCTA images were used for training the DLM. Per vessel (left anterior descending artery (LAD), right coronary artery (RCA), and circumflex artery (Cx)) 9 cMPRs were created at 40° intervals. CorEx analysis was performed fully automated on a remote server using the 9 cMPRs per vessel. A total of 50 CCTAs were uploaded, resulting in CorEx analysis of 150 vessels (LAD, RCA, Cx). The left main was included in the reformatted images of the LAD and the Cx. CorEx output includes a probability score for each CAD-RADS category per vessel and a confidence score for its overall analysis per vessel. The CAD-RADS category with the highest probability score per vessel is considered leading (Supplementary Fig. 1a). However, if the difference in probability score between two adjacent CAD-RADS categories is < 20%, the most severe CAD-RADS category is considered leading (Supplementary Fig. 1b). If CorEx scores its overall analysis with (very) low confidence, the vessel is scored as CAD-RADS category N (Supplementary Fig. 1c).

Two expert European Board of Cardiac Radiology certified CCTA readers also assessed each CCTA set blinded to each other’s results and blinded to CorEx analysis using locally available post-processing software. Reader 1 (RNP) and reader 2 (JFP) with 15 and 20 years of experience in cardiac imaging, respectively. Reader 1 was not involved in training the DLM, while reader 2 was; therefore, the performance of CorEx was compared separately with each reader. Both readers, CorEx-1.0 and CorEx-2.0 classified all 150 vessels using CAD-RADS. Per-vessel CAD-RADS categories were assigned based on the most severe stenosis in that specific vessel. The most severe stenosis in any of the three vessels determines the classification at the patient level. For the binary classification (threshold 70% stenosis), CAD-RADS 0-1-2-3 were merged, separating no to intermediate CAD (CAD-RADS 0-3) from severe CAD (CAD-RADS 4-5).

### Statistical analysis

Continuous data are reported as mean ± standard deviation for normally distributed variables or median with interquartile range (IQR) for non-normally distributed variables. The normality of data distribution was assessed using histograms and probability plots. Categorical data are reported as absolute numbers and percentages. Inter-reader agreement and agreement between CorEx-2.0 and human reading were assessed by calculating the percent agreement, Cohen’s kappa statistic for the binary classification and Cohen’s linearly weighted kappa for the 6-group classification. Kappa values 0.60–0.79 were considered to indicate substantial agreement, and kappa values ≥ 0.80 were considered excellent agreement [24]. The diagnostic performance of reader 2 versus reader 1 (inter-reader analysis) and CorEx-2.0 versus each of the expert readers was evaluated using sensitivity, specificity, positive predictive value (PPV) and negative predictive value (NPV). Patients and vessels assigned with CAD-RADS N were excluded from the analyses. A *p*-value of < 0.05 was considered statistically significant. All statistical analyses were performed using IBM SPSS statistics version 28.

## Results

### Baseline characteristics

The baseline characteristics of the 50 included patients, including the CAD-RADS distribution assigned by reader 1 in clinical practice, are presented in Table 1. The study patients had a mean age of 62.1 years ± 8.3, and 21 (42.0%) were women. An overview of the CAD-RADS categories assigned by both expert readers, CorEx-1.0 and CorEx-2.0 on a per-patient (*n* = 50) and on a per-vessel (*n* = 150) basis is provided in Supplementary Table 1.

**Table 1:** Baseline characteristics

Characteristic	Overall (*n* = 50)
Age, years	62.1 ± 8.3
Female sex	21 (42.0)
Weight, kg	82.3 ± 15.5
BMI, kg/m^2^	26.5 ± 4.1
CAD risk factors	
Diabetes mellitus	8 (16.0)
Hypertension	18 (36.0)
Dyslipidemia	20 (40.0)
Family history of CAD	21 (42.0)
Smoking history	16 (32.0)
Heart rate during CCTA, bpm	59.1 ± 7.0
CAC Agatston score*	158.6 (0.0–330.4)
CAD-RADS score^†^	-
0	5 (10)
1	7 (14)
2	15 (30)
3	9 (18)
4	9 (18)
5	5 (10)
N	-

Continuous data are reported as means ± standard deviations or median with interquartile range (IQR). Categorical data are reported as raw numbers with proportions and percentages

*BMI* denotes body mass index, *bpm* beats per minute, *CAC* coronary artery calcium, *CAD* coronary artery disease, *CAD-RADS* Coronary Artery Disease-Reporting and Data System, *CCTA* coronary computed tomography angiography

* CAC Agatston score was available for 23 of the 50 (46%) patients

^†^ Final CAD-RADS score per patient as assigned in clinical practice by reader 1

### Inter-reader agreement analysis

**Fig. 1:**
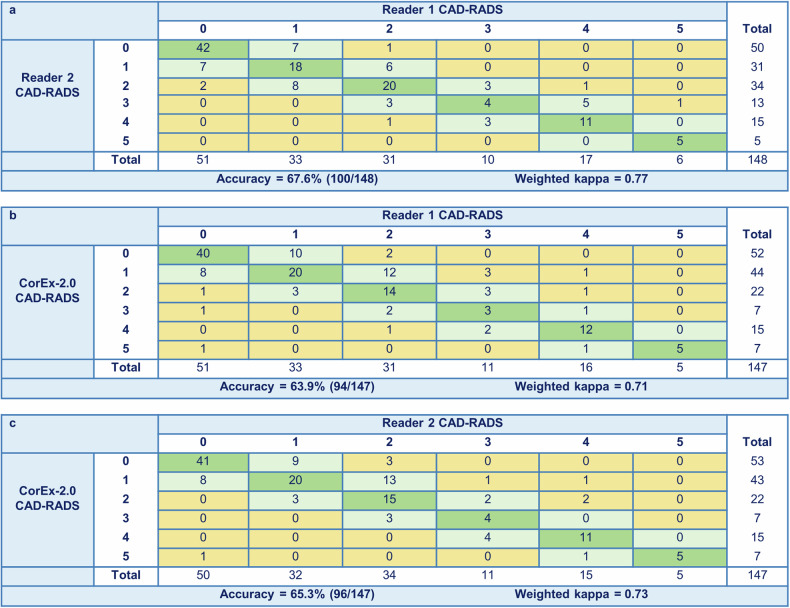
Agreement for the 6-group CAD-RADS classification per vessel. Agreement for the 6-group Coronary Artery Disease-Reporting and Data System (CAD-RADS) classification (CAD-RADS 0-5) per vessel. **a** Agreement per vessel (*n* = 148) between reader 1 and reader 2. **b** Agreement per vessel (*n* = 147) between reader 1 and CorEx-2.0. **c** Agreement per vessel (*n* = 147) between reader 2 and CorEx-2.0. The colors indicate agreement (green), agreement within one category (light green), and disagreement involving more than one category (yellow), respectively

A total of 148 vessels in 50 patients were scored diagnostic image quality by both readers, yielding 49 patients eligible for inter-reader analysis. Agreement between readers and diagnostic performance of reader 2 versus reader 1 for the binary classification threshold of 70% stenosis (CAD-RADS 0-3 versus 4-5) at patient level is reported in Tables 2 and 3. There was disagreement in four patients, resulting in 91.8% (45/49) accuracy and a Cohen’s kappa of 0.80. Inter-reader agreement using the 6-group CAD-RADS classification (CAD-RADS 0-5) per vessel is depicted in Fig. 1a. Agreement on CAD-RADS classification at vessel level between readers using the 6-group classification was 67.6% (100/148) with a weighted kappa of 0.77. Further evaluation of inter-reader agreement using the binary classification (50% (CAD-RADS 0-2 versus 3-5) and 70% (CAD-RADS 0-3 versus 4-5)) at patient and vessel levels is presented in Supplementary Table 2. In addition, inter-reader agreement using the 6-group CAD-RADS classification at patient level is illustrated in Supplementary Fig. 2.

**Table 2:** Agreement using the binary classification (70%) at patient level

Agreement between reader 2 and reader 1				
		**Reader 1**		
Reader 2		< 70% stenosis		≥ 70% stenosis
	< 70% stenosis	33 (33/49; 67.3%)		2 (2/49; 4.1%)
	≥ 70% stenosis	2 (2/49; 4.1%)		12 (12/49; 24.5%)

Agreement between CorEx-2.0 and reader 1				
		**Reader 1**		
CorEx-2.0		< 70% stenosis		≥ 70% stenosis
	< 70% stenosis	33 (33/50; 66.0%)		0 (0/50; 0%)
	≥ 70% stenosis	3 (3/50; 6.0%)		14 (14/50; 28.0%)

Agreement between CorEx-2.0 and reader 2				
		**Reader 2**		
CorEx-2.0		< 70% stenosis		≥ 70% stenosis
	< 70% stenosis	32 (32/49; 65.3%)		0 (0/49; 0%)
	≥ 70% stenosis	3 (3/49; 6.1%)		14 (14/49; 28.6%)

**Table 3:** Diagnostic performance using the binary classification (70%) at patient level

Diagnostic performance of reader 2 with reader 1 as reference							
	**Reader**	**Sensitivity**	**Specificity**	**PPV**	**NPV**	**Accuracy**	**Kappa**
Reader 2	1	85.7% (12/14)	94.3% (33/35)	85.7% (12/14)	94.3% (33/35)	91.8% (45/49)	0.80

Diagnostic performance of CorEx-2.0 with reader 1 and reader 2 as reference							
	**Reader**	**Sensitivity**	**Specificity**	**PPV**	**NPV**	**Accuracy**	**Kappa**
CorEx-2.0	1	100% (14/14)	91.7% (33/36)	82.4% (14/17)	100% (33/33)	94.0% (47/50)	0.86
CorEx-2.0	2	100% (14/14)	91.4% (32/35)	82.4% (14/17)	100% (32/32)	93.9% (46/49)	0.86

*NPV* denotes negative predictive value, *PPV* positive predictive value

### Performance of CorEx-2.0 at patient level

All 50 patients were scored diagnostic image quality by CorEx-2.0, yielding 50 patients (versus reader 1) and 49 patients (versus reader 2) for analysis. Agreement with each of the readers and the diagnostic performance of CorEx-2.0 at patient level was assessed using the binary classification threshold of 70% stenosis (CAD-RADS 0-3 versus 4-5) and is reported in Tables 2 and 3. CorEx-2.0 identified all patients with severe stenosis (CAD-RADS ≥ 4) as classified by human reading, yielding a sensitivity of 100%, accuracy of 94% and Cohen’s kappa values of 0.86 versus reader 1 and reader 2. Further diagnostic performance using the binary classification (threshold 50% stenosis) and using the 6-group CAD-RADS classification at patient level is included as Supplementary material (Supplementary Table 2 and Supplementary Fig. 2). To illustrate the differences in performance between CorEx-1.0 and CorEx-2.0, the performance of CorEx-1.0 at patient level is also presented in Supplementary Table 2 and Supplementary Fig. 2.

### Performance of CorEx-2.0 at vessel level

**Fig. 2:**
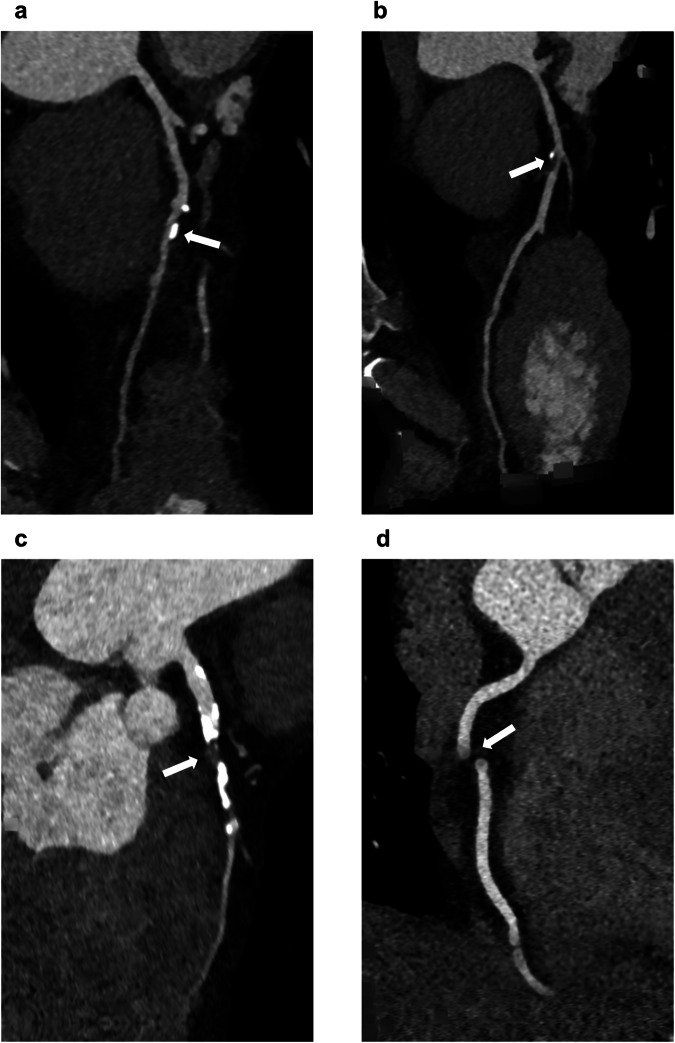
CAD-RADS (dis)agreements between CorEx-2.0 analysis and human reading. Examples of Coronary Artery Disease-Reporting and Data System (CAD-RADS) interpretation agreements and disagreements between CorEx-2.0 analysis and human reading at vessel level. **a** Calcified plaque in the mid left anterior descending artery (LAD) (arrow), classified as CAD-RADS 2 by CorEx-2.0 and CAD-RADS 3 by human. **b** Mixed plaque in the mid LAD (arrow), classified as CAD-RADS 3 by CorEx-2.0 and human. **c** Mixed plaque in the mid LAD (arrow), classified as CAD-RADS 5 by CorEx-2.0 and human. **d** Substantial stair-step artifact in the right coronary artery (arrow), classified as CAD-RADS 5 by CorEx-2.0 and CAD-RADS 0 by human

A total of 147 vessels were eligible for analysis between CorEx-2.0 and expert reading. The performance of CorEx-2.0 at vessel level, compared with each of the expert readers as references, was evaluated using the 6-group CAD-RADS classification (Fig. 1b, c). CorEx-2.0 demonstrated an agreement of 63.9% (94/147) versus reader 1 and 65.3% (96/147) versus reader 2 with weighted kappa values of 0.71 and 0.73, respectively. The most common disagreements were based on underestimation or overestimation of non-obstructive vessels by CorEx-2.0 (*n* = 41 versus reader 1 and *n* = 40 versus reader 2). The most apparent discrepancy was observed in one vessel classified as CAD-RADS 5 by CorEx-2.0, which was identified as CAD-RADS 0 by both readers and was attributable to a substantial stair-step artifact. Examples of (dis)agreements between human reading and CorEx-2.0 analysis are illustrated in Fig. 2. Further diagnostic performance of CorEx-2.0 using the binary classification (threshold 50% and 70% stenosis) at vessel level compared with each of the expert readers as references is presented in Supplementary Table 2. The diagnostic performance of CorEx-1.0 at vessel level is also presented as Supplementary material (Supplementary Table 2 and Supplementary Fig. 3). The change in CAD-RADS score after updating from CorEx-1.0 to CorEx-2.0 is illustrated in Supplementary Fig. 4.

## Discussion

This single-center retrospective study evaluated the diagnostic performance of a recently updated DLM (CorEx-2.0) for quantifying coronary stenosis on CCTA, using a binary CAD-RADS classification threshold of 70% stenosis (CAD-RADS 0-3 versus CAD-RADS 4-5) per patient and a 6-group CAD-RADS classification (CAD-RADS 0-5) per-vessel. CorEx-2.0 identified all patients with severe stenosis (CAD-RADS ≥ 4), yielding 100% sensitivity and 94% accuracy for the binary classification, while also demonstrating good performance using the 6-group classification per vessel (weighted kappa values > 0.70), both compared separately with two independent expert CCTA readers as references.

In a previous study, the pre-updated model (CorEx-1.0) achieved 96% accuracy for detecting ≥ 50% stenosis at patient level among 53 patients with predominantly non-obstructive CAD (72%) [20]. However, CorEx-1.0 demonstrated lower accuracy (84%) in 217 patients presenting with acute chest pain [25]. The updated model (CorEx-2.0) recently achieved 82% accuracy for detecting ≥ 50% stenosis in a high-risk population (62.7% obstructive CAD on CCTA) during transcatheter aortic valve replacement workup [22]. These previous studies were limited to the binary (CAD-RADS 0-2 versus CAD-RADS 3-5) or 3-group CAD-RADS classification (CAD-RADS 0, 1-2, or 3-4-5). In our validation study, the CAD-RADS distribution within the study population was more balanced, and a 6-group CAD-RADS classification per vessel was assigned by both CorEx-2.0 and the expert readers. This 6-group analysis is a more accurate method compared with the binary or 3-group analysis used in the previous studies. We further focused on the analysis of the binary classification (CAD-RADS 0-3 versus CAD-RADS 4-5) at patient level, which represents a threshold for medical therapy with potential additional interventional treatment (≥ 70% stenosis) [8]. Nearly two-thirds of the patients with ≥ 70% stenosis (CAD-RADS ≥ 4) undergo revascularization [26]. The performance characteristics of CorEx-2.0 in our study compare similarly or favorably to those reported in other studies [17–19, 23, 27, 28]. However, it should be mentioned that a direct comparison with the listed studies is not feasible, as different study populations with varying sizes and CAD-RADS distributions were used. One single-center retrospective study compared an in-house DLM with expert readers in 288 patients and demonstrated 71% accuracy for the binary classification (threshold 50% stenosis) at patient level [27]. Zreik et al achieved 85% accuracy for this binary analysis in 65 patients by comparing their research method with an expert reader [23]. These studies did not perform a binary classification (threshold 70% stenosis) or 6-group CAD-RADS analysis. Regarding this binary classification (threshold 50%) at patient level, CorEx-2.0 performed better than or similarly to their models, yielding an accuracy of 82% versus reader 1 and 90% versus reader 2. A multicenter study by Choi et al enrolled 232 patients and used a commercial AI model that identified all patients with severe stenosis (≥ 70%) versus consensus reading, similar to the performance of CorEx-2.0 [17]. On a per-vessel basis, the model showed weighted kappa values of 0.69, 0.57, and 0.67 versus individual expert readers for the 6-group CAD-RADS classification, which is lower than the performance of CorEx-2.0 versus expert readers (weighted kappa of 0.71 versus reader 1 and 0.73 versus reader 2). The weighted kappa of 0.72 at vessel level compared with consensus reading, as demonstrated by Choi et al, is comparable to our results. Notably, only 5% of the patients and only 1.5% of the vessels were classified as CAD-RADS ≥ 4 (≥ 70% stenosis), indicating a low disease prevalence in their study population. This may limit the ability to adequately assess the safety of this tool in detecting severe stenosis (≥ 70%). Another study evaluated the same AI model compared with invasive quantitative coronary angiography in a cohort of 303 patients with a higher disease prevalence (≥ 70% stenosis in 39% of patients and 19% of vessels), demonstrating a per-patient sensitivity and accuracy of 94% and 86%, respectively, for detecting ≥ 70% stenosis [28]. In our study, 28% of the patients and 15% of the vessels were classified as CAD-RADS ≥ 4 (≥ 70% stenosis) by expert reading, and CorEx-2.0 yielded 100% sensitivity and 94% accuracy. However, these results are difficult to compare, as we used expert CCTA readers as references. Moreover, their study did not conduct a 6-group CAD-RADS analysis per vessel. This 100% detection of ≥ 70% stenosis at patient level compared with expert readers, as achieved by CorEx-2.0, was also established by Lin and colleagues using a novel research DLM in 50 patients [18]. At vessel level, this DLM demonstrated a Cohen’s kappa of 0.78 compared to expert CCTA interpretation using a 5-group CAD-RADS classification (CAD-RADS 1-5). However, since a 6-group CAD-RADS analysis was not performed, it is difficult to compare their results with the weighted kappa values of 0.71 (versus reader 1) and 0.73 (versus reader 2) per vessel using the 6-group classification presented in our study. Recently, Van Herten et al designed a fully automated deep learning-based method for plaque segmentation and CAD-RADS grading [19]. This research model achieved a linearly weighted kappa of 0.71 for the 6-group CAD-RADS classification per patient in an external test set of 658 patients, which aligns with our results for the 6-group classification at patient level (weighted kappa of 0.67 versus reader 1 and 0.74 versus reader 2). However, their test set had a significantly lower disease prevalence (only 4.6% of patients with CAD-RADS ≥ 4), which may have influenced the model’s performance. Furthermore, despite good performance, their method tended to overestimate CAD severity [19]. These potential false-positive results may lead to unnecessary downstream testing [29], raising concerns about the significantly increased utilization of CCTA. With respect to CorEx-2.0, a marginal underestimation compared with human assessment was observed, yet the model proved to be safe for identifying patients with severe stenosis compared with a fully independent expert reader. These findings underscore the potential of CorEx-2.0 to flag patients at risk of severe obstructive CAD.

This study had several limitations. First, the retrospective single-center design and the exclusion of patients with non-diagnostic examinations or a history of coronary interventions could have resulted in selection bias. Second, the CAD-RADS distribution in the study population may not reflect local clinical practice; this might have influenced the performance of CorEx-2.0, and this holds implications for direct comparison with other studies for local performance and reproducibility of the presented results. Therefore, further studies with larger patient cohorts are needed to evaluate CorEx-2.0 performance in a more standard outpatient cardiology CCTA population. Third, one of the expert readers trained the DLM, which could induce bias for a one-to-one comparison with CorEx-2.0. However, the other expert reader (reader 1) showed similar performance and was not involved in the DLM training and development. Fourth, only the three main vessels (LAD, RCA, Cx) were included in this study. Finally, invasive coronary angiography was not performed routinely, and therefore, we could not adequately assess the performance of both expert readers in this subset of cases.

## Conclusion

In this study, the updated DLM (CorEx-2.0) identified all patients with severe stenosis (CAD-RADS ≥ 4) when compared separately with two independent expert readers, yielding a sensitivity of 100% for detecting severe stenosis. CorEx-2.0 also demonstrated good performance approaching expert readers for the 6-group CAD-RADS classification at vessel level (weighted kappa > 0.70). These findings highlight the potential for CorEx-2.0 to be safely integrated into clinical practice, where it could be helpful in prioritizing CCTA reading by flagging patients at risk of severe obstructive coronary artery disease.

## Supplementary information

ELECTRONIC SUPPLEMENTARY MATERIAL

## Abbreviations

AI: Artificial intelligence
CAD: Coronary artery disease
CAD-RADS: Coronary Artery Disease-Reporting and Data System
CCTA: Coronary computed tomography angiography
Cx: Circumflex
DLM: Deep learning model
LAD: Left anterior descending
NPV: Negative predictive value
PPV: Positive predictive value
RCA: Right coronary artery
