# Supplementary material for: Automated classification of coronary LEsions fRom coronary computed Tomography angiography scans with an updated deep learning model: ALERT study
Source: Eur Radiol. 2025 Jan 10;35(3):1543–51. doi: 10.1007/s00330-024-11308-z (PMC11836176; doi:10.1007/s00330-024-11308-z)
Supplement: Supplementary file 1 — ELECTRONIC SUPPLEMENTARY MATERIAL [file 330_2024_11308_MOESM1_ESM.pdf]

Automated classification of coronary LEsions fRom coronary computed Tomography angiography scans with an updated deep learning model: ALERT study

ELECTRONIC SUPPLEMENTARY MATERIAL

Supplemental Table 1. CAD-RADS categories assigned by human, CorEx-1.0 and CorEx-2.0

**Per-patient (n = 50)**					
**CAD-RADS, n (%)**	**Reader 1**	**Reader 2**	**CorEx-1.0**	**CorEx-2.0**	**P-value***
**0**	5 (10.0%)	6 (12.0%)	0 (0.0%)	5 (10.0%)	0.06
**1**	7 (14.0%)	7 (14.0%)	2 (4.0%)	14 (28.0%)	0.004
**2**	15 (30.0%)	13 (26.0%)	14 (28.0%)	9 (18.0%)	0.36
**3**	9 (18.0%)	9 (18.0%)	14 (28.0%)	5 (10.0%)	0.02
**4**	9 (18.0%)	9 (18.0%)	7 (14.0%)	10 (20.0%)	0.25
**5**	5 (10.0%)	5 (10.0%)	7 (14.0%)	7 (14.0%)	1.00
**N**	0 (0.0%)	1 (2.0%)	6 (12.0%)	0 (0.0%)	0.03
**Mean score**	2.50	2.47	3.07	2.44	<0.001
**Per-vessel (n = 150)**					
**CAD-RADS, n (%)**	**Reader 1**	**Reader 2**	**CorEx-1.0**	**CorEx-2.0**	**P-value***
**0**	51 (34.0%)	50 (33.3%)	20 (13.3%)	53 (35.3%)	< 0.001
**1**	33 (22.0%)	32 (21.3%)	29 (19.3%)	44 (29.3%)	0.04
**2**	31 (20.7%)	34 (22.7%)	43 (28.7%)	22 (14.7%)	0.005
**3**	11 (7.3%)	13 (8.7%)	23 (15.3%)	7 (4.7%)	<0.001
**4**	17 (11.3%)	15 (10.0%)	13 (8.7%)	15 (10.0%)	0.75
**5**	6 (4.0%)	5 (3.3%)	7 (4.7%)	7 (4.7%)	1.00
**N**	1 (0.7%)	1 (0.7%)	15 (10.0%)	2 (1.3%)	<0.001
**Mean score**	1.52	1.50	2.01	1.38	<0.001

CAD-RADS denotes Coronary Artery Disease-Reporting and Data System.
* P-value to compare differences in percentages per CAD-RADS category and mean CAD-RADS score CorEx-1.0 and CorEx-2.0.
McNemar tests were used to compare the percentages per CAD-RADS category between CorEx-1.0 and CorEx-2.0.
The Wilcoxon signed-rank test was used to test the differences in mean CAD-RADS scores between CorEx-1.0 and CorEx-2.0.

Supplemental Table 2. Diagnostic performance using the binary classification (50% and 70%)

Diagnostic performance of reader 2 with reader 1 as reference (binary classification 50%)								
	Basis	***Reader***	***Sensitivity***	***Specificity***	***PPV***	***NPV***	***Accuracy***	***Kappa***
Reader 2	Patient-level	1	86.4% (19/22)	85.2% (23/27)	82.6% (19/23)	88.5% (23/26)	85.7% (42/49)	0.71
	Vessel-level	1	87.9% (29/33)	96.5% (111/115)	87.9% (29/33)	96.5% (111/115)	94.6% (140/148)	0.84
Diagnostic performance of reader 2 with reader 1 as reference (binary classification 70%)								
	Basis	***Reader***	***Sensitivity***	***Specificity***	***PPV***	***NPV***	***Accuracy***	***Kappa***
Reader 2	Patient-level	1	85.7% (12/14)	94.3% (33/35)	85.7% (12/14)	94.3% (33/35)	91.8% (45/49)	0.80
	Vessel-level	1	69.6% (16/23)	96.8% (121/125)	80.0% (16/20)	94.5% (121/128)	92.6% (137/148)	0.70
Diagnostic performance of CorEx-1.0 with human reading as reference (binary classification 50%)								
	Basis	***Reader***	***Sensitivity***	***Specificity***	***PPV***	***NPV***	***Accuracy***	***Kappa***
CorEx-1.0	Patient-level	1	100% (20/20)	66.7% (16/24)	71.4% (20/28)	100% (16/16)	81.8% (36/44)	0.65
		2	100% (21/21)	69.6% (16/23)	75.0% (21/28)	100% (16/16)	84.1% (37/44)	0.69
	Vessel-level	1	93.3% (28/30)	85.6% (89/104)	65.1% (28/43)	97.8% (89/91)	87.3% (117/134)	0.68
		2	96.6% (28/29)	85.8% (91/106)	65.1% (28/43)	98.9% (91/92)	88.1% (119/135)	0.70
Diagnostic performance of CorEx-1.0 with human reading as reference (binary classification 70%)								
	Basis	***Reader***	***Sensitivity***	***Specificity***	***PPV***	***NPV***	***Accuracy***	***Kappa***
CorEx-1.0	Patient-level	1	85.7% (12/14)	93.3% (28/30)	85.7% (12/14)	93.3% (28/30)	90.9% (40/44)	0.79
		2	92.3% (12/13)	93.5% (29/31)	85.7% (12/14)	96.7% (29/30)	93.2% (41/44)	0.84
	Vessel-level	1	76.2% (16/21)	96.5% (109/113)	80.0% (16/20)	95.6% (109/114)	93.3% (125/134)	0.74
		2	94.4% (17/18)	97.4% (114/117)	85.0% (17/20)	99.1% (114/115)	97.0% (131/135)	0.88
Diagnostic performance of CorEx-2.0 with human reading as reference (binary classification 50%)								
	Basis	***Reader***	***Sensitivity***	***Specificity***	***PPV***	***NPV***	***Accuracy***	***Kappa***
CorEx-2.0	Patient-level	1	78.3% (18/23)	85.2% (23/27)	81.8% (18/22)	82.1% (23/28)	82.0% (41/50)	0.64
		2	87.0% (20/23)	92.3% (24/26)	90.9% (20/22)	88.9% (24/27)	89.8% (44/49)	0.80
	Vessel-level	1	75.0% (24/32)	95.7% (110/115)	82.8% (24/29)	93.2% (110/118)	91.2% (134/147)	0.73
		2	80.6% (25/31)	96.6% (112/116)	86.2% (25/29)	94.9% (112/118)	93.2% (137/147)	0.79
Diagnostic performance of CorEx-2.0 with human reading as reference (binary classification 70%)								
	Basis	***Reader***	***Sensitivity***	***Specificity***	***PPV***	***NPV***	***Accuracy***	***Kappa***
CorEx-2.0	Patient-level	1	100% (14/14)	91.7% (33/36)	82.4% (14/17)	100% (33/33)	94.0% (47/50)	0.86
		2	100% (14/14)	91.4% (32/35)	82.4% (14/17)	100% (32/32)	93.9% (46/49)	0.86
	Vessel-level	1	85.7% (18/21)	96.8% (122/126)	81.8% (18/22)	97.6% (122/125)	95.2% (140/147)	0.81
		2	85.0% (17/20)	96.1% (122/127)	77.3% (17/22)	97.6% (122/125)	94.6% (139/147)	0.78

NPV denotes negative predictive value, PPV positive predictive value.

Supplemental Figure 1. Examples of CorEx-2.0 analyses and interpretation

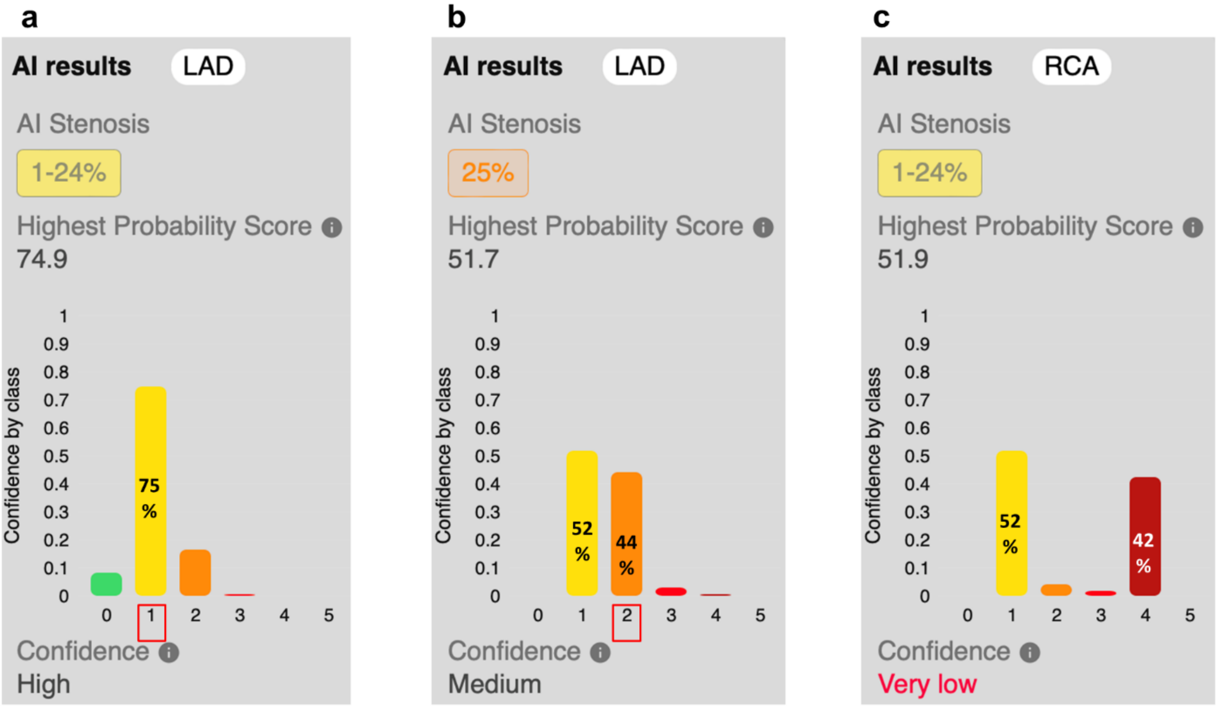

Figure legend:
Examples of CorEx-2.0 analyses and interpretation. **a** CAD-RADS 1 is considered leading as it is the CAD-RADS category assigned by CorEx-2.0 with the highest probability score. **b** CAD-RADS 2 is considered leading as the difference in probability score between two adjacent CAD-RADS categories is <20% assigned by CorEx-2.0, so the most severe CAD-RADS category (CAD-RADS 2) is considered leading. **c** CorEx-2.0 scored ’very low’ confidence, so the vessel is scored as CAD-RADS category N.

Supplemental Figure 2. Agreement for the 6-group CAD-RADS classification per-patient

Per patient		Reader 1 CAD-RADS						
		0	1	2	3	4	5	Total
Reader 2 CAD-RADS	0	4	2	0	0	0	0	6
	1	1	3	3	0	0	0	7
	2	0	2	8	3	0	0	13
	3	0	0	3	4	2	0	9
	4	0	0	1	1	7	0	9
	5	0	0	0	0	0	5	5
	Total	5	7	15	8	9	5	49
Accuracy = 63.3% (31/49)				Weighted kappa = 0.77				

Per patient		Reader 1 CAD-RADS						
		0	1	2	3	4	5	Total
CorEx-1.0 CAD-RADS	0	0	0	0	0	0	0	0
	1	1	1	0	0	0	0	2
	2	2	5	7	0	0	0	14
	3	0	0	6	6	2	0	14
	4	0	0	1	0	6	0	7
	5	1	0	0	0	1	5	7
	Total	4	6	14	6	9	5	44
Accuracy = 56.8% (25/44)				Weighted kappa = 0.61				

Per patient		Reader 1 CAD-RADS						
		0	1	2	3	4	5	Total
CorEx-2.0 CAD-RADS	0	2	3	0	0	0	0	5
	1	2	4	6	2	0	0	14
	2	0	0	6	3	0	0	9
	3	0	0	2	3	0	0	5
	4	0	0	1	1	8	0	10
	5	1	0	0	0	1	5	7
	Total	5	7	15	9	9	5	50
Accuracy = 56.0% (28/50)				Weighted kappa = 0.67				

Per patient		Reader 2 CAD-RADS						
		0	1	2	3	4	5	Total
CorEx-1.0 CAD-RADS	0	0	0	0	0	0	0	0
	1	2	0	0	0	0	0	2
	2	1	7	6	0	0	0	14
	3	0	0	6	7	1	0	14
	4	0	0	0	1	6	0	7
	5	1	0	0	0	1	5	7
	Total	4	6	12	8	8	5	44
Accuracy = 54.5% (24/44)				Weighted kappa = 0.71				

Per patient		Reader 2 CAD-RADS						
		0	1	2	3	4	5	Total
CorEx-2.0 CAD-RADS	0	3	1	1	0	0	0	5
	1	2	6	5	1	0	0	14
	2	0	0	6	2	0	0	8
	3	0	0	1	4	0	0	5
	4	0	0	0	2	8	0	10
	5	1	0	0	0	1	5	7
	Total	6	7	13	9	9	5	49
Accuracy = 65.3% (32/49)				Weighted kappa = 0.74				

Figure legend:
Agreement for the 6-group Coronary Artery Disease-Reporting and Data System (CAD-RADS) classification (CAD-RADS 0-5) per-patient. The colors indicate agreement (green), agreement within one category (light green), and disagreement involving more than one category (yellow), respectively.

Supplemental Figure 3. Agreement for the 6-group CAD-RADS classification per-vessel

Per vessel		Reader 1 CAD-RADS						
		0	1	2	3	4	5	Total
Reader 2 CAD-RADS	0	42	7	1	0	0	0	50
	1	7	18	6	0	0	0	31
	2	2	8	20	3	1	0	34
	3	0	0	3	4	5	1	13
	4	0	0	1	3	11	0	15
	5	0	0	0	0	0	5	5
	Total	51	33	31	10	17	6	148
Accuracy = 67.6% (100/148)				Weighted kappa = 0.77				

Per vessel		Reader 1 CAD-RADS						
		0	1	2	3	4	5	Total
CorEx-1.0 CAD-RADS	0	16	3	1	0	0	0	20
	1	17	7	5	0	0	0	29
	2	15	14	11	2	0	0	42
	3	0	2	11	5	5	0	23
	4	0	0	1	2	10	0	13
	5	1	0	0	0	1	5	7
	Total	49	26	29	9	16	5	134
Accuracy = 40.3% (54/134)				Weighted kappa = 0.54				

Per vessel		Reader 1 CAD-RADS						
		0	1	2	3	4	5	Total
CorEx-2.0 CAD-RADS	0	40	10	2	0	0	0	52
	1	8	20	12	3	1	0	44
	2	1	3	14	3	1	0	22
	3	1	0	2	3	1	0	7
	4	0	0	1	2	12	0	15
	5	1	0	0	0	1	5	7
	Total	51	33	31	11	16	5	147
Accuracy = 63.9% (94/147)				Weighted kappa = 0.71				

Per vessel		Reader 2 CAD-RADS						
		0	1	2	3	4	5	Total
CorEx-1.0 CAD-RADS	0	19	1	0	0	0	0	20
	1	14	11	4	0	0	0	29
	2	13	15	14	1	0	0	43
	3	0	0	14	8	1	0	23
	4	0	0	0	2	11	0	13
	5	1	0	0	0	1	5	7
	Total	47	27	32	11	13	5	135
Accuracy = 50.4% (68/135)				Weighted kappa = 0.62				

Per vessel		Reader 2 CAD-RADS						
		0	1	2	3	4	5	Total
CorEx-2.0 CAD-RADS	0	41	9	3	0	0	0	53
	1	8	20	13	1	1	0	43
	2	0	3	15	2	2	0	22
	3	0	0	3	4	0	0	7
	4	0	0	0	4	11	0	15
	5	1	0	0	0	1	5	7
	Total	50	32	34	11	15	5	147
Accuracy = 65.3% (96/147)				Weighted kappa = 0.73				

Figure legend:
Agreement for the 6-group Coronary Artery Disease-Reporting and Data System (CAD-RADS) classification (CAD-RADS 0-5) per-vessel. The colors indicate agreement (green), agreement within one category (light green), and disagreement involving more than one category (yellow), respectively.

Supplemental Figure 4. CAD-RADS score reclassification from CorEx-1.0 to CorEx-2.0

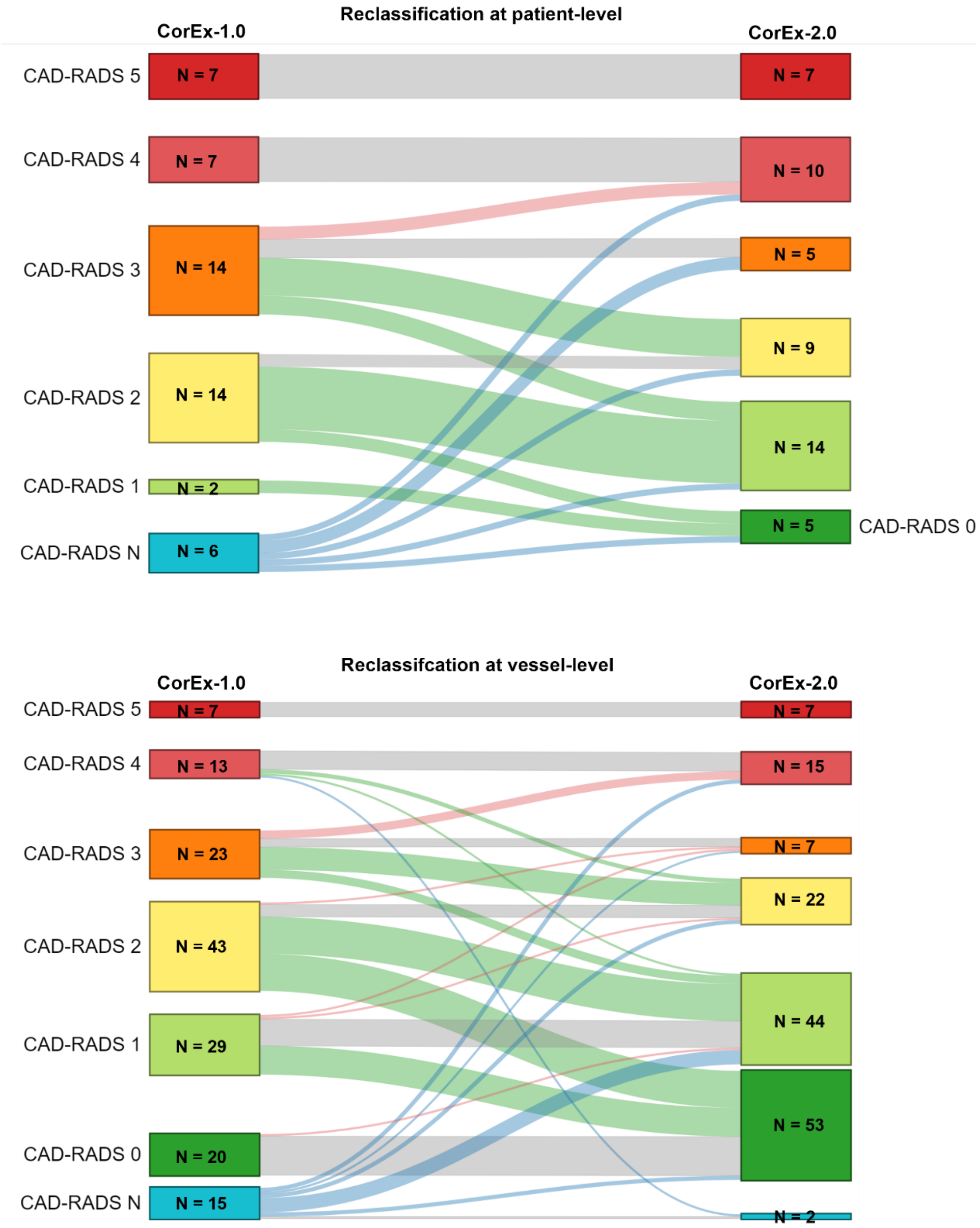

Figure legend:
N = number of patients. Sankey diagram demonstrates the Coronary Artery Disease-Reporting and Data System (CAD-RADS) score reclassification from CorEx-1.0 to CorEx-2.0. The colored flows indicate patients/vessels without change (grey), reclassification to a higher CAD-RADS score (red) by CorEx-2.0, reclassification to a lower CAD-RADS score by CorEx-2.0 (green), and reclassification with CAD-RADS N involved (blue), respectively.
